# Randomized Trial on Echocardiography-Guided Ductus Arteriosus Treatment to Reduce Necrotizing Enterocolitis

**DOI:** 10.3389/fped.2021.807712

**Published:** 2022-01-28

**Authors:** María Carmen Bravo, Rebeca Sánchez-Salmador, María Teresa Moral-Pumarega, Manuela López-Azorín, Rocío Mosqueda-Peña, Izaskun Dorronsoro, Fernando Cabañas, Adelina Pellicer

**Affiliations:** ^1^Department of Neonatology, Hospital Universitario La Paz, Madrid, Spain; ^2^Department of Neonatology, Hospital Universitario 12 de Octubre, Madrid, Spain; ^3^Department of Neonatology, Hospital Universitario Quirónsalud, Madrid, Spain

**Keywords:** ibuprofen, necrotizing enterocolitis, ductus arteriosus, preterm, echocardiographically-guided, Neonatologist Performed Echocardiography

## Abstract

**Objective:**

Patent ductus arteriosus (PDA) approach remains controversial. We aim to evaluate whether echocardiography-guided (EchoG) PDA closure (to reduce drug exposure) and 24-h continuous ibuprofen infusion (24 h-IB) (to reduce peak concentration), compared with EchoG PDA closure plus conventional bolus (bolus-IB), reduces severe bowel adverse event rate in preterm infants with hemodynamically significant (hs) PDA.

**Study Design:**

The study design is a multicenter, blinded, randomized controlled trial. Infants with <28 weeks of gestation underwent routine echocardiographic assessment (18–72 h of birth); infants with 28–33 weeks were screened only in cases where PDA was clinically suspected. HsPDA was considered if ductal diameter >1.5 mm and indicators of pulmonary overflow, systemic hypoperfusion, or both were present. Pharmacodynamic effect of CYP450 genotypes was also analyzed.

**Results:**

One hundred forty-six infants [median gestational age 26 (25–28) weeks; median birth weight 881 (704–1,100) g] were randomized to 24 h-IB (*n* = 70) or bolus-IB (*n* = 76) study group at 86 (58–140) h from birth. Groups were comparable regarding perinatal and neonatal clinical data, but higher prevalence of male sex in the bolus-IB group was found. Neither severe bowel adverse event rate [10% (24 h-IB) and 2.6% (bolus-IB), *p* = 0.1] nor ductal closure rate was different between the study groups. Postnatal age and peripheral SaO2 at treatment start and pulmonary hemorrhage were associated with severe bowel events, independent of treatment group allocation. CYP2C8 genetic polymorphisms were associated with ibuprofen efficacy (*p* = 0.03).

**Conclusions:**

Ibuprofen intravenous continuous infusion compared with bolus infusion in preterm infants with hsPDA shows similar rates of success and does not reduce the prevalence of severe bowel events.

## Introduction

Many controversies concerning patent ductus arteriosus (PDA) approach remain in the last decade. Active PDA treatment is usually decided when there is consensus on its hemodynamic significance (hs), expressed as high risk of causing disease related to pulmonary overflow and systemic hypoperfusion. However, the best treatment option should address both, effectiveness and safety.

Although ibuprofen has been pointed out as the drug of choice (1), necrotizing enterocolitis (NEC) or spontaneous intestinal perforation (SIP) have been observed in 6–12% of the preterm infants who received this treatment (2–4). Conventional therapy consists in three intravenous ibuprofen doses (10, 5, and 5 mg/kg) 24 h apart, delivered in 15 min (1). More recent research suggested greater effectiveness of continuous ibuprofen intravenous infusion and trends toward lower NEC rate (5).

Echocardiography-guided (EchoG) PDA closure has been used for closing PDA with indomethacin (6, 7) and ibuprofen (1) showing that this approach is feasible, reaches the same rates of success, and reduces the number of ibuprofen doses that the infant receives (1, 2).

Finally, pharmacogenetics may be important determinants of ibuprofen bioavailability (therapeutic failure related with quick clearance and higher rate of adverse effects related to lower clearance) (8, 9). This pharmacogenetic information is critical in personalized medicine, when only a given drug should be administered, i.e., ibuprofen, if successful treatment rate overcomes the potential rates of side effects.

We hypothesized that in the preterm infant with an hsPDA, a treatment scheme consistent with EchoG PDA closure (to reduce drug exposure) immediately followed by 24-h continuous ibuprofen infusion (to reduce peak concentrations of ibuprofen), compared with EchoG PDA closure plus conventional bolus ibuprofen treatment, reduces NEC and SIP prevalence. As a secondary objective, we aim to address whether other factors influence the study outcomes.

## Materials and Methods

This trial was conducted at three level 3C neonatal intensive care units of the Madrid region from May 2017 to July 2020.

### Eligibility Criteria

Infants with <28 completed weeks of gestation underwent routine echocardiographic assessment between 18 and 72 h of birth; those infants between 28 and 33 weeks of gestation were screened only if there was a clinical suspicion of PDA. HsPDA was defined in the case of ductal diameter larger than 1.5 mm and the presence of indicators of pulmonary overflow [antegrade left pulmonary artery (LPA) diastolic flow >30 cm/s or left atrial: aortic ratio >1.5 or LVO/SVC flow ratio ≥4; or E wave/A wave ratio ≥1], systemic hypoperfusion (absent or reverse diastolic velocity in the postductal aorta), or both; following the latest recommendations of the European Special Interest Group “Neonatologist Performed Echocardiography” (10). The information on eligible infants for the study was transferred to their respective attending physician who made the decision on PDA treatment or not, based on echocardiographic criteria and the clinical condition. If an open duct was not judged as hsPDA, or even in the case of pulmonary overflow or systemic hypoperfusion, the attending physician decided a “watchful waiting; ” serial scans were undertaken to confirm PDA closure or need for treatment prescription.

After informed consent and enrollment, infants were randomly allocated to two ibuprofen treatment schemes: ibuprofen continuous infusion (24 h-IB) or ibuprofen standard bolus infusion (bolus-IB). The exclusion criteria were contraindications to ibuprofen treatment (renal insufficiency, severe intracranial bleeding, bowel complications, or pulmonary hypertension), life-threatening defects, congenital heart disease, or refused consent. Whether a formal contraindication for ibuprofen was observed, but it was decided to close the PDA, iv paracetamol was administered as treatment.

### Echocardiographic Studies

An Aplio 500 (Toshiba Medical Systems BV, Netherlands), an Acuson X300 (Siemens, Germany), and a LOGIQ S8 XDclear 2.0 (General Electric, USA) scanners were used to establish structural normality of the heart and the hemodynamic significance of PDA (10). The transductal diameter, ductal maximum and minimum velocity (Vmax/Vmin) ratio, antegrade left pulmonary artery (LPA) diastolic flow, left atrial/aortic ratio, left ventricular output (LVO), right cardiac output (RVO), superior vena cava (SVC) flow, and E wave/A wave ratio and diastolic velocity in the postductal aorta were obtained.

### Intervention

Ten milligrams per kilogram per day of ibuprofen (Pedea^®^ 10 mg/2 ml) was administered either in continuous infusion during 24 h or in 15 min infusion bolus. Two additional 5 mg/kg doses were scheduled every 24 h, following the same scheme whether PDA remained open 24 h after each dose. Twenty-four h after the first trial of ibuprofen (one to three doses), an echocardiography was repeated, and subsequent doses were planned if needed up to six. In the case hsPDA persisted after two ibuprofen courses, and there was difficulty to wean from the ventilator, and protracted metabolic acidosis or persistent hemodynamic instability were present, surgical PDA closure was proposed in accordance with the pediatric cardiology team.

### Randomization and Blinding

A website was used for centralized randomization, taking into account gestational age (<28 weeks and ≥28 weeks) and center (Scientific Support Unit, Hospital Doce Octubre). A numerical list with a random allocation sequence was generated to identify the sealed envelope where the treatment group assigned to each participant was described. The sealed envelope with the study identification number was delivered to a research nurse that was not involved in the care of the patient who prepared the study medication. For each infant, two identical syringes, identified as “bolus” and “continuous infusion,” were prepared. Infants randomized to “24 h-IB” received a bolus of placebo consisting of 2 ml/kg of glucose 5% in 15 min immediately followed by a continuous solution for infusion of ibuprofen (2 ml/kg diluted in glucose 5%) at weight-adjusted infusion rate (0.1 or 0.2 ml/h in infants below 1,200 g or above 1,201 g, respectively). Infants randomized to “bolus-IB” received a bolus of the corresponding dose of ibuprofen (2 ml/kg) in 15 min immediately followed by a continuous infusion of placebo (glucose 5%) at the same infusion rate as described above. In the case where additional doses were prescribed according to EchoG, the same prescription protocol was used.

### Outcome Measures

Perinatal, main neonatal diagnoses at term-equivalent age or discharge (whatever came first), hemodynamic and echocardiographic parameters were recorded in an electronic database specifically designed for this purpose. The primary outcome measure, adverse bowel event (Bell stage 2 or more of NEC or SIP), was defined as the presence of intestinal pneumatosis, portal venous gas, or pneumoperitoneum in an abdominal x-ray study. Treatment successful rate was defined as ductus echocardiographic closure confirmation 24 h after the last dose of ibuprofen. PDA reopening was defined as the presence of a duct that needed additional treatment (pharmacological or surgical) after echocardiographic closure confirmation. Structural brain injury was defined by blinded operators according to cranial ultrasound (CUS) findings, following the latest recommendations of the EurUSbrain group (11–13). The first CUS was performed at enrollment; during admission, subsequent exams were done based on individual findings, but routine exams every 2 weeks and at term equivalent or before discharge were always conducted. The most severe cranial ultrasound diagnoses in a given infant throughout the whole observation period was considered for analyses.

### Genetics

After inclusion, DNA was collected from the epithelial cells of the infant with a cotton swab applied gently to both cheeks and immediately sent to the INGEMM (Institute of Medical and Molecular Genetics, La Paz Hospital) for analysis. Buccal swab DNA was isolated using High Pure PCR Template Preparation Kit (Roche, Switzerland) according to the protocol of the manufacturer. DNA obtained was stored at −20°C until processing. Samples were classified according to the enzymatic activity in the following genotypes: NM, normal/extensive metabolizers; IM, intermediate metabolizers; PM, poor metabolizers, and UM, ultrarapid metabolizers.

### Statistics

This trial was powered to demonstrate a reduction of NEC or SIP from 9% (global incidence of NEC in preterm infants treated with ibuprofen for closing PDA) (2–4) to 0.1% (published incidence of NEC during ibuprofen continuous infusion treatment for closing PDA) (5) with a power of 80% and *p* < 0.05; thus, the calculated sample size was 180 infants (90 infants in each study arm). To assess the secondary outcome, potential associations between perinatal, hemodynamic, respiratory, CYP enzyme genotypes, or echocardiographic variables and primary outcomes (NEC or SIP) were also examined.

Quantitative variables were expressed as median and interquartile range (IQR), and qualitative data as count and percentage (%). Comparisons between study groups were done using the Mann–Whitney U or Kruskal–Wallis test and Fisher's exact or χ^2^ test for quantitative and qualitative variables, respectively [SAS version 9.4 (SAS Institute Inc., 2013)].

All the statistical analyses considered bilateral *p*-values < 0.05 as significant.

The study protocol (14) was reviewed and approved by the Ethics Committee for Human Studies at La Paz University Hospital and the Spanish Medicines Agency.

## Results

**Figure 1:**
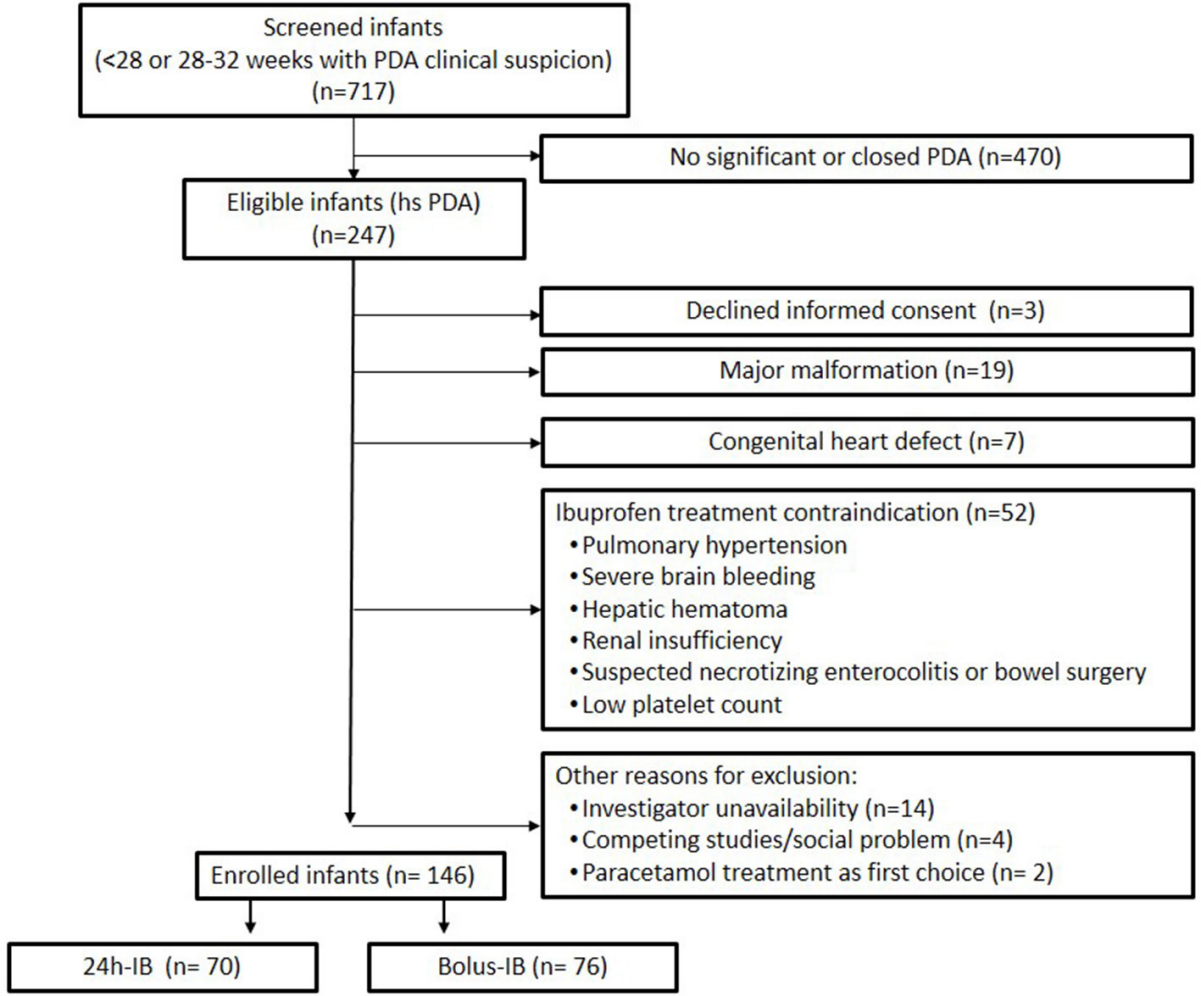
Patient flowchart.

During 38 months of study period, 717 preterm infants below 33 weeks of gestation were considered eligible; among them, 146 with 26 (25–28) weeks of gestation and 881 (704–1,100) g of birth weight were enrolled and randomized to 24 h-IB (*n* = 70) or bolus-IB (*n* = 76) (Figure 1). The inclusion rate was lower than expected due to more restricted criteria for treating PDA with respect to the time when the trial was planned. The trial was closed after 3 years of enrollment.

Groups did not differ with respect to perinatal and neonatal clinical data, with the exception of male sex, which was more prevalent in the bolus-IB group (*p* = 0.004; Table 1).

**Table 1:** Clinical data of the study population.

	**24 h-IB**	**Bolus-IB**	***P*** **-value**
	**(***n*** = 70)**	**(***n*** = 76)**	
Gestational age (weeks), median (IQR)	26 (25–27)	26 (25–29)	0.6
Birth weight (g), median (IQR)	850 (702–990)	900 (706–1153)	0.2
Maternal age (years), median (IQR)	34 (30.2–38)	33 (30–35)	0.4
5-min Apgar score <5 (IQR)	7 (6–8)	8 (6–8)	0.2
Cord pH, median (IQR)	7.31 (7.14–7.35)	7.31 (7.25–7.36)	0.4
Premature rupture of membranes >24 h, *n* (%)	10 (14.5)	13 (17.1)	0.8
Advanced resuscitation, *n* (%)*	44 (63)	47 (63)	1
SNAPPE II, median (IQR)^#^	32 (18.5–49)	31 (17–43)	0.3
Chorioamnionitis, *n* (%)^†^	28 (42)	34 (48)	0.6
Antenatal steroids, *n* (%)^z^	67 (96)	69 (92)	0.6
Multiple, *n* (%)	29 (41)	33 (44)	0.9
Male sex, *n* (%)	25 (35)	46 (61)	0.004
SGA, *n* (%)	10 (14)	7 (9)	0.5
Postnatal age at “before treatment” Echocardiography, (hours) median (IQR)	91 (62–137)	73 (47–138)	0.1
Postnatal age at start ibuprofen treatment (hours), median (IQR)	94 (67–139)	78 (55–140)	0.2
Platelet count (× 10^3^/ml) before ibuprofen treatment, median (IQR)	185 (132–294)	203 (146–270)	0.4
Hemoglobin (g/dL) before ibuprofen treatment, median (IQR)	13.6 (12–15)	13.1 (12–14.7)	0.3
Creatinine (mg/dL) before ibuprofen treatment, median (IQR)	0.8 (0.6–0.9)	0.8 (0.7–0.9)	0.5
Protein C reactive (mg/L) before ibuprofen treatment, median (IQR)	2.4 (0.3–5)	2.9 (0.5–7.8)	0.4
Ibuprofen doses administered (median, IQR)	5 (2–6)	4 (2–6)	0.4

* *Intubation in the delivery room with or without assisted circulation*.

# *SNAPPE-II, Score for Neonatal Acute Physiology Perinatal Extension-II (19)*.

† *Histological placental studies*.

z *Full course. SGA, small for gestational age (birth weight <3rd centile)*.

The echocardiography before treatment was conducted at 83.5 (52.2–138) h of postnatal life, and ibuprofen treatment started at 86 (58–140) h from birth. Treatment success was confirmed in 52% of the infants [24 h-IB (51%), bolus-IB (54%), *p* = 0.9] who showed no ductal flow after ibuprofen treatment. Median total number of ibuprofen doses administered was 5 (2–6), without differences between groups (Table 1). In 35 of the enrolled infants, PDA closure was documented after single or two ibuprofen doses. PDA reopening did not differ between study groups (seven infants by group), and five of these infants underwent surgical ligation. Ibuprofen treatment was halted in 26 infants (24 h-IB = 13, bolus-IB = 12, *p* = 0.8) due to potential treatment-related adverse events (intracranial bleeding, platelet count <50 × 10^3^, suspected NEC, pulmonary hypertension, or renal insufficiency). Cardiorespiratory status and echocardiographic parameters were evaluated, considering before and after treatment periods, which are described in Table 2.

**Table 2:** Evolution of cardiorespiratory status and echocardiographic variables during the intervention.

	**Before treatment**			**After treatment**			**Before treatment**	**After treatment**	
	**24 h-IB** **(***n*** = 70)**	**Bolus-IB** **(***n*** = 76)**	***P*** **-value**	**24 h-IB** **(***n*** = 70)**	**Bolus-IB** **(***n*** = 76)**	***P*** **-value**	**Full cohort** **(***n*** = 146)**	**Full cohort** **(***n*** = 146)**	***P*** **-value**
Transductal diameter, mm (median, IQR)	2.5 (2.1–3)	2.4 (2.2–2.8)	0.6	0 (0–1.5)	0 (0–1.4)	0.8	2.5 (2.1–2.9)	0 (0–1.4)	<0.001
Ductal velocity Vmax/Vmin ratio (median, IQR)	2 (1.6–2.6)	1.9 (1.5–2.3)	0.07	* Vmax: 0 (0–1) Vmin: 0 (0–0.2)	* Vmax: 0 (0–9) Vmin: 0 (0–0.4)	*	2 (1.6–2.4)	* Vmax: 0 (0–1) Vmin: 0 (0–0.3)	*
Antegrade LPA diastolic flow, cm/s (median, IQR)	37 (30–45)	36 (30–44)	0.9	13 (8–27)	13 (10–27)	0.6	36 (30–44)	13 (8–27)	<0.001
Left atrial: aortic ratio (median, IQR)	1.8 (1.6–2)	2 (1.7–2.2)	0.08	1.6 (1.4–1.8)	1.6 (1.4–1.8)	0.9	1.9 (1.7–2.1)	1.6 (1.4–1.8)	0.5
LVO (median, IQR)	240 (209–318)	249 (202–285)	0.5	206 (181–248)	215 (164–255)	0.8	247 (206–291)	209 (175–250)	<0.001
RVO (median, IQR)	314 (261–461)	274 (202–352)	0.1	326 (228–414)	264 (227–373)	0.3	287 (239–394)	295 (228–393)	0.6
SVC flow (median, IQR)	89 (68–122)	85 (62–110)	0.2	114 (78–150)	103 (80–132)	0.5	87 (64–118)	104 (79–143)	0.001
E wave/A wave ratio (median, IQR)	0.8 (0.7–1)	0.8 (0.7–1)	0.1	0.8 (0.7–0.9)	0.8 (0.7–0.9)	0.9	0.8 (0.7–1)	0.8 (0.7–0.9)	0.02
Reverse or absent descending aorta diastolic velocity, *n* (%)	55 (84)	47 (68)	0.04	5 (10)	7 (13)	0.7	102 (70)	13 (9)	<0.001
Heart rate, bpm (median, IQR)	155 (146–165)	157 (148–162)	0.9	158 (148–166)	155 (145–164)	0.3	157 (147–164)	157 (147–165)	0.9
Systolic blood pressure, mmHg (median, IQR)	54 (48–62)	53 (45–60)	0.2	58 (50–65)	55 (50–65)	0.6	53 (46–61)	56 (50–65)	0.007
Diastolic blood pressure, mmHg (median, IQR)	29 (23–37)	37 (32–44)	0.9	33 (27–41)	31 (27–42)	0.6	29 (24–37)	32 (27–41)	0.002
Mean airway pressure, cmH2O (median, IQR)	7.8 (5.1–10)	6.3 (5–8.9)	0.3	7.7 (6–9.4)	8.2 (6–11)	0.5	7.7 (5.8–9.8)	7.8 (6–10)	0.5
Lactic acid, mmol/L (median, IQR)	1.5 (1–2)	1.4 (0.9–2)	0.5	1 (0.8–1.5)	1 (0.8–1.9)	0.3	1.5 (1.1–2.1)	1 (0.8–1.7)	<0.001
Base excess (median, IQR), mmol/L	−2.6 [−5.3 to (−0.9)]	−2.2 [−4.8 to (−0.9)]	0.3	−0.9 (−2.4 to 1.2)	−0.4 (−3.1 to 2.3)	0.8	−2.7 [−5.2 to (−0.5)]	−0.8 (−2.7 to 2)	<0.001
Urine output, ml/kg/min (median, IQR)	3.8 (3.1–4.6)	3.6 (3–4.4)	0.3	2.8 (2.1–3.8)	3 (2.3–3.7)	0.5	3.7 (3.1–4.5)	2.9 (2.2–3.7)	<0.001

* *Undefined. 24 h-IB, Ibuprofen continuous infusion; bolus-IB, Ibuprofen standard bolus infusion. IQR, interquartile range; LPA, left pulmonary artery; LVO, left ventricular output; RVO, right ventricular output; SCV, superior vena cava; Vmax, maximum velocity; Vmin, minimum velocity*.

Seven infants died, and NEC or SIP was diagnosed in nine infants, without differences between the study groups. There were no differences regarding other neonatal outcomes with the exception of time to reach full enteral nutrition that was achieved earlier in the bolus-IB group (*p* = 0.03) (Table 3).

**Table 3:** Main neonatal diagnosis at term equivalent age.

	**24 h-IB**	**Bolus-IB**	***P*** **-value**
	**(***n*** = 70)**	**(***n*** = 76)**	
Mortality *n* (%)	1 (1.4)	6 (8)	0.1
NEC or SIP, *n* (%)	7 (10)	2 (2.6)	0.1
Combined outcome: mortality or NEC or SIP, *n* (%)	8 (11.4)	7 (9.2)	0.8
Normal CUS or IVH grade I, *n* (%)	41 (59)	24 (33)	0.4
IVH grade II or III, *n* (%)	8 (11)	14 (19)	0.3
PVIH, *n* (%)	1 (1.4)	3 (4.1)	0.6
White matter damage, *n* (%)	16 (23)	18 (25)	0.9
Cerebellum injury, *n* (%)	1 (1.4)	4 (5.4)	0.4
Full enteral feeds, days (median, IQR)	19.5 (12.2–28.7)	14 (10–25)	0.03
PDA reopening after confirmed closed, *n* (%)	7 (10)	7 (9)	1
Surgical closure of PDA, *n* (%), *n* (%)	22 (31)	16 (21)	0.2
O_2_ dependency at 36 weeks, *n* (%)	39 (56)	42 (60)	0.8
ROP treatment, *n* (%)	16 (32)	10 (14)	0.8
Age at discharge, days (median, IQR)	88 (68–104)	85.5 (58–99)	0.12

*NEC, necrotising enterocolitis; SIP, spontaneous intestinal perforation; CUS, cranial ultrasound; IVH, intraventricular hemorrhage; PVIH, periventricular hemorrhagic infarction; PDA, patent ductus arteriosus; ROP, retinopathy of prematurity*.

The association between relevant perinatal and neonatal clinical data, as well as the hemodynamic status with the study primary outcome (NEC or SIP) was explored. A higher postnatal age (0.02) and lower peripheral SaO2 (0.04) immediately before PDA treatment, as well as a diagnosis of pulmonary hemorrhage during the first 2 weeks of life (0.03), were associated with the risk of developing NEC or SIP, independent of group allocation (Table 4).

**Table 4:** Associations between adverse bowel outcomes and perinatal, clinical or echocardiographic features in the study population.

	**NEC or SIP absent**	**NEC or SIP present**	***P*** **-value**
	**(***n*** = 137)**	**(***n*** = 9)**	
Gestational age (weeks), median (IQR)	26 (25–28)	25 (24–27)	0.1
Birth weight (g), median (IQR)	880 (711–1120)	805 (620–980)	0.2
5–min Apgar score <5, (IQR)	7 (6–8)	7 (7–8)	0.9
Advanced resuscitation, *n* (%)*	84 (61)	8 (89)	0.2
Chorioamnionitis, *n* (%)^†^	58 (44)	5 (62)	0.5
Antenatal steroids, *n* (%)^z^	128 (93)	9 (100)	0.9
Multiple, *n* (%)	59 (43)	3 (33)	0.8
Male sex, *n* (%)	68 (50)	4 (44)	1
SGA, *n* (%)	15 (11)	2 (22)	0.6
Age at treatment start (hours), median (IQR)	82 (58–134)	125 (105–156)	0.02
Number of ibuprofen doses, median (IQR)	5 (2–6)	3 (2–6)	0.6
Platelet count (x 10^3^/ml) before treatment, median (IQR)	212 (108)	217 (131)	0.9
Hemoglobin (g/dL) before treatment, median (IQR)	14.4 (10.8)	13.4 (1.8)	0.8
Peripheral SaO_2_ at treatment start (%), median (IQR)	96 (94–98)	92 (90–93)	0.04
Mean airway pressure, at treatment start (cmH_2_O), median (IQR)	7 (5–9.6)	7.2 (5.2–8.4)	0.6
FiO_2_, at treatment start (%), median (IQR)	21 (21–27)	26.5 (22.5–30.7)	0.1
Pulmonary hemorrhage before treatment, *n* (%)	9 (7)	3 (33)	0.03
Transductal diameter (mm), median (IQR)	2.5 (2.2–2.9)	2.1 (2–2.4)	0.5
Ductal velocity (Vmax/Vmin) ratio (m/s), median (IQR)	1.9 (1.5–2.4)	2.5 (2–3.4)	0.1
Antegrade LPA diastolic flow (cm/s), median (IQR)	36 (30–44)	40 (38–42)	0.2
Left atrial: aortic ratio, median (IQR)	1.9 (0.3)	1.8 (0.3)	0.4
LVO (mL/Kg/min), median (IQR)	259 (76)	246 (53)	0.6
RVO (mL/Kg/min), median (IQR)	343 (173)	348 (130)	0.9
SVC flow (mL/Kg/min), median (IQR)	98 (51)	83 (23)	0.5
E wave/A wave ratio, median (IQR)	0.8 (0.7–1)	0.8 (0.8–0.9)	0.6
Reverse o absent descending aorta diastolic velocity, *n* (%)	111 (87)	6 (100)	0.7
Mother's own or donor milk, *n* (%)	132 (96)	9 (100)	1
Probiotics, *n* (%)	20 (15)	2 (22)	0.9

* *Intubation in the delivery room with or without assisted circulation*.

# *SNAPPE-II, Score for Neonatal Acute Physiology Perinatal Extension-II (19)*.

† *Histological placental studies*.

z *Full course. SGA, small for gestational age (birth weight <3rd centile)*.

One hundred and thirty-four samples were studied for the CYP450 genotypes. Twelve epithelial cell samples were discarded due to technical issues. Infants with normal CYP2C8 enzymatic activity showed lower treatment successful rate (*p* = 0.03). None of the genotypes were associated with safety (Table 5).

**Table 5:** Effect of CYP450 genotypes on pharmacodynamics.

**Genotype data**		**Pharmacodynamic variables**				
**CYP450 enzimes**	**Enzymatic activity**	**Cohort analyzed**	**Treatment success**	***P*** **-value**	**NEC or SIP or death**	***P*** **-value**
		***n*** **= 134**	***n*** **= 68**	***n*** **= 13**	
CYP2C8	NM	81	36	0.03	11	0.3
	IM	27	15		0	
	PM	5	3		0	
	Not amplify	12	5		1	
	Indetermined	9	9		1	
CYP2C9	NM	94	45	0.3	9	0.7
	IM	7	3		0	
	Not amplify	26	14		3	
	Indetermined	7	6		1	
CYP2C19	NM	47	23	0.3	6	0.7
	IM	2	1		0	
	UM	1	1		0	
	Not amplify	78	38		7	
	Indetermined	6	5		0	
CYPC3a4	NM	108	51	0.5	11	0.3
	IM	5	3		0	
	Not amplify	13	8		0	
	Indetermined	8	6		2	

*NM, normal/extensive metabolizers; IM, intermediate metabolizers; PM, poor metabolizers; UM, ultrarapid metabolizers*.

## Discussion

This trial has not shown a reduction in the incidence of severe adverse bowel events after continuous intravenous ibuprofen infusion compared with standard treatment. In addition, the experimental treatment did not show benefit in terms of effectiveness. These findings are not in agreement with a previous trial where the continuous infusion showed trends toward lower NEC prevalence and higher closure rates than the conventional treatment (5). There are some reasons that may explain this discrepancy. First, the infants in the present study were more immature and with lower birth weight. Second, the definition of NEC differed between Lago's and this study, as they considered any stage of NEC occurring during treatment, while in the present study, NEC is defined as Bell's stage 2 or above throughout the whole admission. In addition, in that study, the power calculation was done to demonstrate effectiveness and not to demonstrate a reduction in NEC. In addition, the definition of hsPDA deserving treatment changed from the time when the study was planned to the onset of the beginning of recruitment (ibuprofen only administered in case of low chance of early spontaneous closure and echocardiographic signs of pulmonary overflow or systemic hypoperfusion). This approach has shown to be useful to build a predictive model on early spontaneous PDA closure and, consequently, in the selection of infants that may have a greater benefit to participate in intervention trials on the prevention of PDA-related morbidities (15). According to this policy, only one third of the eligible cohort was classified as having an hsPDA, therefore, accounting for the lower rate of PDA successful treatment.

The use of EchoG treatment in this study prevented unnecessary ibuprofen doses in one in four patients, without an increase in the reopening rate (10%) compared with that reported with the classical scheme (12%) (1). The rationale to use EchoG treatment and continuous infusion of ibuprofen was to reduce drug exposure and the peak concentration of ibuprofen, respectively (16). Although the risk of NEC associated to ibuprofen compared with indomethacin treatment appears lower (1), and the total dose was reduced in this trial, severe bowel complications occurred in 6% of the full cohort. The pathophysiology of NEC is multifactorial (17). We explored the parameters traditionally associated with PDA and their relationship with the onset of NEC. Lower peripheral oxygen saturation immediately before treatment, more advanced postnatal age at treatment, and pulmonary hemorrhage before treatment were more frequent in the subgroup of infants who developed NEC than in those who did not. These findings are in agreement with the hypothesis that infants with NEC might be exposed to a more prolonged effect of PDA with lower systemic perfusion and pulmonary overflow. However, the number of patients who developed adverse bowel outcome was too low to build a predictive model. Human milk was universally administered to all the participating infants, but probiotics were very rarely administered, thus, explaining the lack of discriminative beneficial effect. Although severe anemia during the first postnatal week is a risk factor of NEC (18), in this trial, neither hemoglobin concentration nor platelet count at the start of treatment was associated with bowel adverse event rates.

Finally, genetic variations in ibuprofen metabolism were explored. It was observed that the genotypes with poor or intermediate CYP2C8 enzymatic activity showed higher PDA closure rate than infants with normal activity. Thus, CYP450 genotype polymorphisms seem to play a role in the interindividual variability of ibuprofen effectiveness. As all the pharmacogenetic studies were performed on buccal epithelial cells collected with a cotton swab, this study highlights that this non-invasive technique is feasible in the preterm population.

This study has several limitations. First, the planned sample size was not reached. However, after the inclusion of 81% of the full cohort, the analysis did not show any trend favoring the experimental treatment. Thus, even if the planned enrollment had been accomplished, the hypothesis of reducing the rate of adverse bowel events in the 24 h-IB would not have been achieved. Second, the pharmacogenetic analyses may not be extrapolated to other populations as they were performed in a small number of infants. Nevertheless, the genotype CYP2C8^*^1/^*^3 plus CYP2C9^*^1/^*^2 was found in 11% of our study population, which is in agreement with rates found in the Spanish population that shows 19% prevalence for this mutation (9). This finding supports that the sample may represent the global population in our setting.

In conclusion, EchoG treatment for hsPDA with intravenous ibuprofen continuous infusion, compared with intravenous ibuprofen bolus, does not reduce the rate of adverse bowel events in preterm infants. Other strategies should be explored to improve the safety during PDA treatment.

## Data Availability Statement

The data are not publicly available due to this analysis containing information that could compromise the privacy of research participants, but the data presented in this study are available on request from the corresponding author (Dr. María Carmen Bravo; mcarmen.bravo@salud.madrid.org).

## Ethics Statement

The studies involving human participants were reviewed and approved by Ethics Committee for Human Studies at La Paz University Hospital and the Spanish Medicines Agency. Written informed consent to participate in this study was provided by the participants' legal guardian/next of kin.

## Author Contributions

MB conceptualized and designed the study, drafted the initial manuscript, collected the information from the medical charts, performed the initial analyses, and approved the final manuscript as submitted. RS-S, MM-P, ML-A, RM-P, and ID collected the information from the medical charts and approved the final manuscript as submitted. FC conceptualized and designed the study and approved the final manuscript as submitted. AP conceptualized and designed the study, drafted the initial manuscript, and approved the final manuscript as submitted. All authors contributed to the article and approved the submitted version.

## Funding

This study was supported by the Spanish Health Ministry (PI16/00644) and the Mutua Madrileña Fundation (AP163272016).

## Conflict of Interest

The authors declare that the research was conducted in the absence of any commercial or financial relationships that could be construed as a potential conflict of interest.

## Publisher's Note

All claims expressed in this article are solely those of the authors and do not necessarily represent those of their affiliated organizations, or those of the publisher, the editors and the reviewers. Any product that may be evaluated in this article, or claim that may be made by its manufacturer, is not guaranteed or endorsed by the publisher.

## Abbreviations

NEC: necrotizing enterocolitis
SIP: spontaneous intestinal perforation
PDA: patent ductus arteriosus
Iv: intravenous
EchoG: echocardiography guided
hsPDA: hemodynamically significant PDA
24 h-IB: ibuprofen continuous infusion
bolus-IB: ibuprofen standard bolus infusion.
